# Supplementary material for: Determination of the Structure and Dynamics of the Fuzzy Coat of an Amyloid Fibril of IAPP Using Cryo-Electron Microscopy
Source: Biochemistry. 2023 Jul 21;62(16):2407–16. doi: 10.1021/acs.biochem.3c00010 (PMC10433526; doi:10.1021/acs.biochem.3c00010)
Supplement: Supplementary file 1 — bi3c00010_si_001.pdf [file bi3c00010_si_001.pdf]

Supplementary Information

Determination of the structure and dynamics of the fuzzy coat of an amyloid fibril of IAPP using cryo-electron microscopy

Z. Faidon Brotzakis[1,‡], Thomas Löhr[1, ‡], Steven Truong[1], Samuel Hoff[2],
Massimiliano Bonomi[2] and Michele Vendruscolo[1,*]

[1]Centre for Misfolding Diseases, Department of Chemistry,
University of Cambridge, Cambridge CB2 1EW, UK
[2]Institut Pasteur, Université Paris Cité, CNRS UMR 3528,
Department of Structural Biology and Chemistry, 75015 Paris, France

‡ Authors have contributed equally
** Corresponding author: mv245@cam.ac.uk*

A

Left side N-terminal tail

Description	Plumed file notation	Manuscript Notation	Biased
Torsion dihedral of disulfide bond CyS2-4 of peptide 1	disulf1	χ_3^1 (MEMMI)	Yes
Torsion dihedral of disulfide bond CyS2-4 of peptide 2	disulf2	χ_3^2 (MEMMI)	Yes
Torsion dihedral of disulfide bond CyS2-4 of peptide 3	disulf3	χ_3^3 (MEMMI)	Yes
Torsion dihedral of disulfide bond CyS2-4 of peptide 4	disulf4	χ_3^4 (MEMMI)	Yes
Torsion dihedral of disulfide bond CyS2-4 of peptide 5	disulf5	χ_3^5 (MEMMI)	Yes
Torsion dihedral of disulfide bond CyS2-4 of peptide 6	disulf6	χ_3^6 (MEMMI)	Yes
Torsion dihedral of disulfide bond CyS2-4 of peptide 7	disulf7	χ_3^7 (MEMMI)	Yes
Torsion dihedral of disulfide bond CyS2-4 of peptide 8	disulf8	χ_3^8 (MEMMI)	Yes
Twist around peptide 1 axis	tor1	t^1 (MEMMI)	Yes
Twist around peptide 2 axis	tor2	t^2 (MEMMI)	Yes
Twist around peptide 3 axis	tor3	t^3 (MEMMI)	Yes
Twist around peptide 4 axis	tor4	t^4 (MEMMI)	Yes
Twist around peptide 5 axis	tor5	t^5 (MEMMI)	Yes
Twist around peptide 6 axis	tor6	t^6 (MEMMI)	Yes
Twist around peptide 7 axis	tor7	t^7 (MEMMI)	Yes
Twist around peptide 8 axis	tor8	t^8 (MEMMI)	Yes
Contacts between chain 1-2	con1	$N_{1\text{-}2}$ (MEMMI)	Yes
Contacts between chain 2-3	con2	$N_{2\text{-}3}$ (MEMMI)	Yes
Contacts between chain 3-4	con3	$N_{3\text{-}4}$ (MEMMI)	Yes
Contacts between chain 4-5	con4	$N_{4\text{-}5}$ (MEMMI)	Yes
Contacts between chain 5-6	con5	$N_{5\text{-}6}$ (MEMMI)	Yes
Contacts between chain 6-7	con6	$N_{6\text{-}7}$ (MEMMI)	Yes
Contacts between chain 7-8	con7	$N_{7\text{-}8}$ (MEMMI)	Yes

Right side N-terminal tail

Description	Plumed file notation	Manuscript Notation	Biased
Torsion dihedral of disulfide bond CyS2-4 of peptide 1	disulf9	χ_3^1 (EMMI)	No
Torsion dihedral of disulfide bond CyS2-4 of peptide 2	disulf10	χ_3^2 (EMMI)	No
Torsion dihedral of disulfide bond CyS2-4 of peptide 3	disulf11	χ_3^3 (EMMI)	No
Torsion dihedral of disulfide bond CyS2-4 of peptide 4	disulf12	χ_3^4 (EMMI)	No
Torsion dihedral of disulfide bond CyS2-4 of peptide 5	disulf13	χ_3^5 (EMMI)	No
Torsion dihedral of disulfide bond CyS2-4 of peptide 6	disulf14	χ_3^6 (EMMI)	No
Torsion dihedral of disulfide bond CyS2-4 of peptide 7	disulf15	χ_3^7 (EMMI)	No
Torsion dihedral of disulfide bond CyS2-4 of peptide 8	disulf16	χ_3^8 (EMMI)	No
Twist around peptide 1 axis	tor9	t^1 (EMMI)	No
Twist around peptide 2 axis	tor10	t^2 (EMMI)	No
Twist around peptide 3 axis	tor11	t^3 (EMMI)	No
Twist around peptide 4 axis	tor12	t^4 (EMMI)	No
Twist around peptide 5 axis	tor13	t^5 (EMMI)	No
Twist around peptide 6 axis	tor14	t^6 (EMMI)	No
Twist around peptide 7 axis	tor15	t^7 (EMMI)	No
Twist around peptide 8 axis	tor16	t^8 (EMMI)	No
Contacts between chain 1-2	con8	$N_{1\text{-}2}$ (EMMI)	No
Contacts between chain 2-3	con9	$N_{2\text{-}3}$ (EMMI)	No
Contacts between chain 3-4	con10	$N_{3\text{-}4}$ (EMMI)	No
Contacts between chain 4-5	con11	$N_{4\text{-}5}$ (EMMI)	No
Contacts between chain 5-6	con12	$N_{5\text{-}6}$ (EMMI)	No
Contacts between chain 6-7	con13	$N_{6\text{-}7}$ (EMMI)	No
Contacts between chain 7-8	con14	$N_{7\text{-}8}$ (EMMI)	No

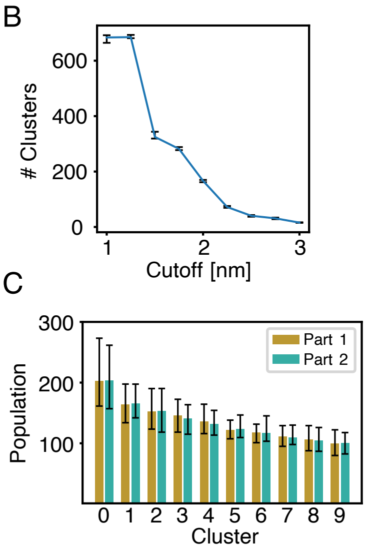

Figure S1. Metadynamics and clustering information. (A) Summary of all biased collective variables of the left side N-terminal tail and the respective unbiased ones on the right side N-terminal tail used in the analysis. **(B)** Dependence of number of clusters on the cut-off value used in the GROMOS clustering algorithm, using root-mean-square deviations of Cα atoms in tails 3 and 4 of the left (biased) side. **(C)** Populations of the top 10 clusters for the two last 40 % chunks of the simulation. Error bars for **(B)** and **(C)** show the 95th percentiles over 20 separate clustering runs using 5000 frames sampled based on metadynamics weights from each part of the simulation.

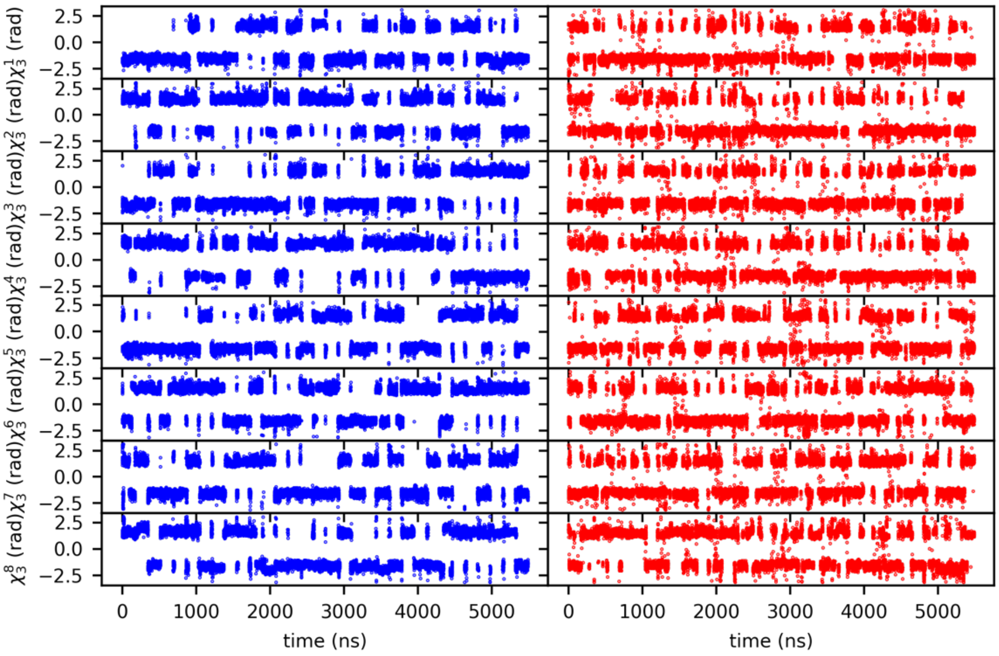
χ_3^1 (rad)
χ_3^2 (rad)
χ_3^3 (rad)
χ_3^4 (rad)
χ_3^5 (rad)
χ_3^6 (rad)
χ_3^7 (rad)
χ_3^8 (rad)
time (ns)
time (ns)

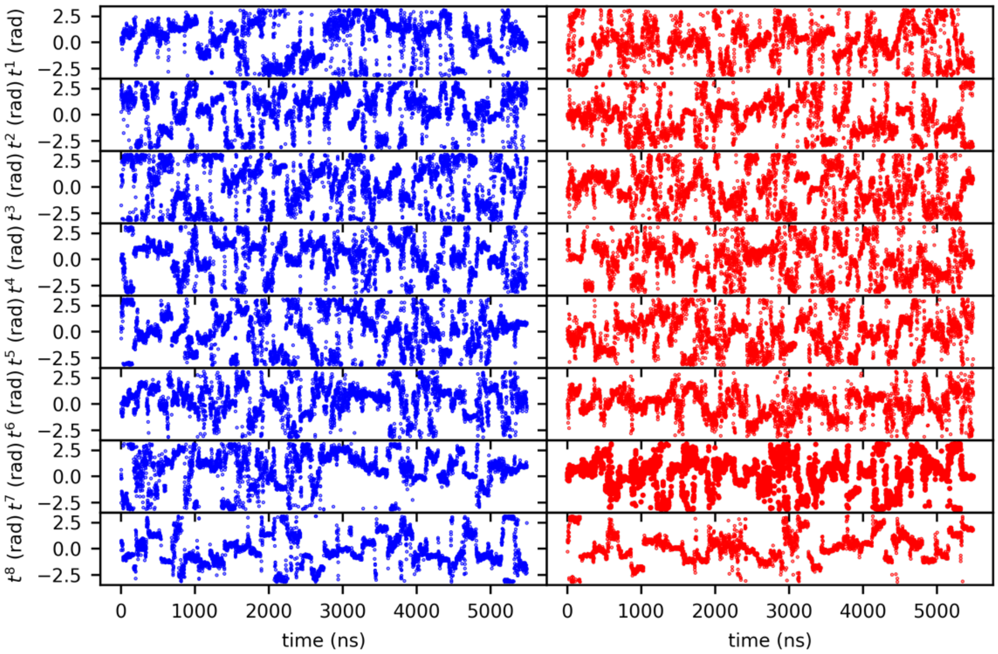
t^1 (rad)
t^2 (rad)
t^3 (rad)
t^4 (rad)
t^5 (rad)
t^6 (rad)
t^7 (rad)
t^8 (rad)
time (ns)
time (ns)

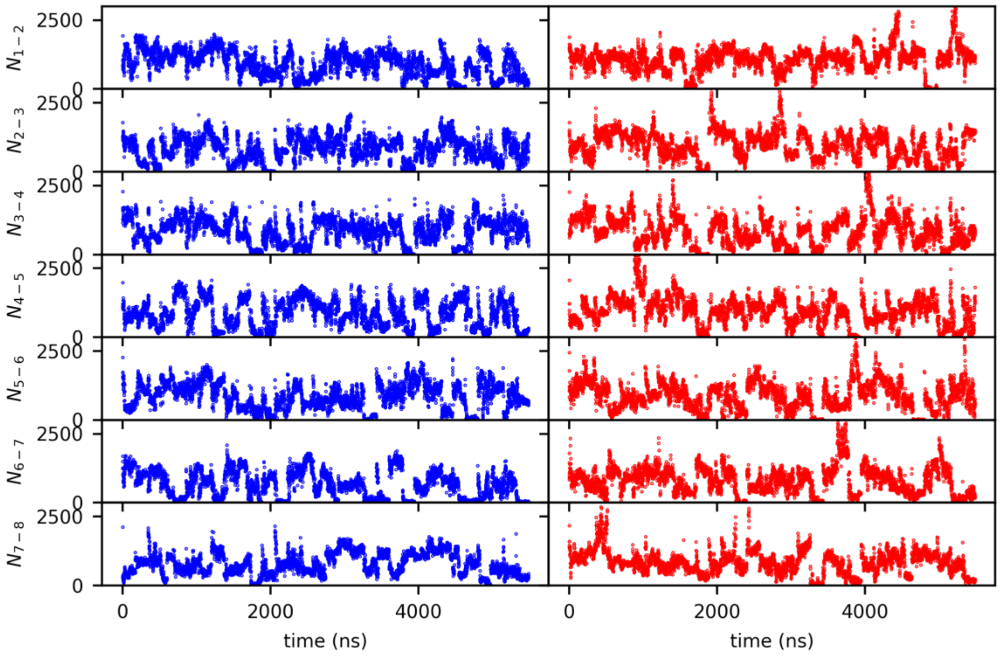

Figure S2. Assessment of the convergence of the simulations MEMMI-EMMI. Time evolution profiles for all unbiased (blue) and respective biased CVs (red).

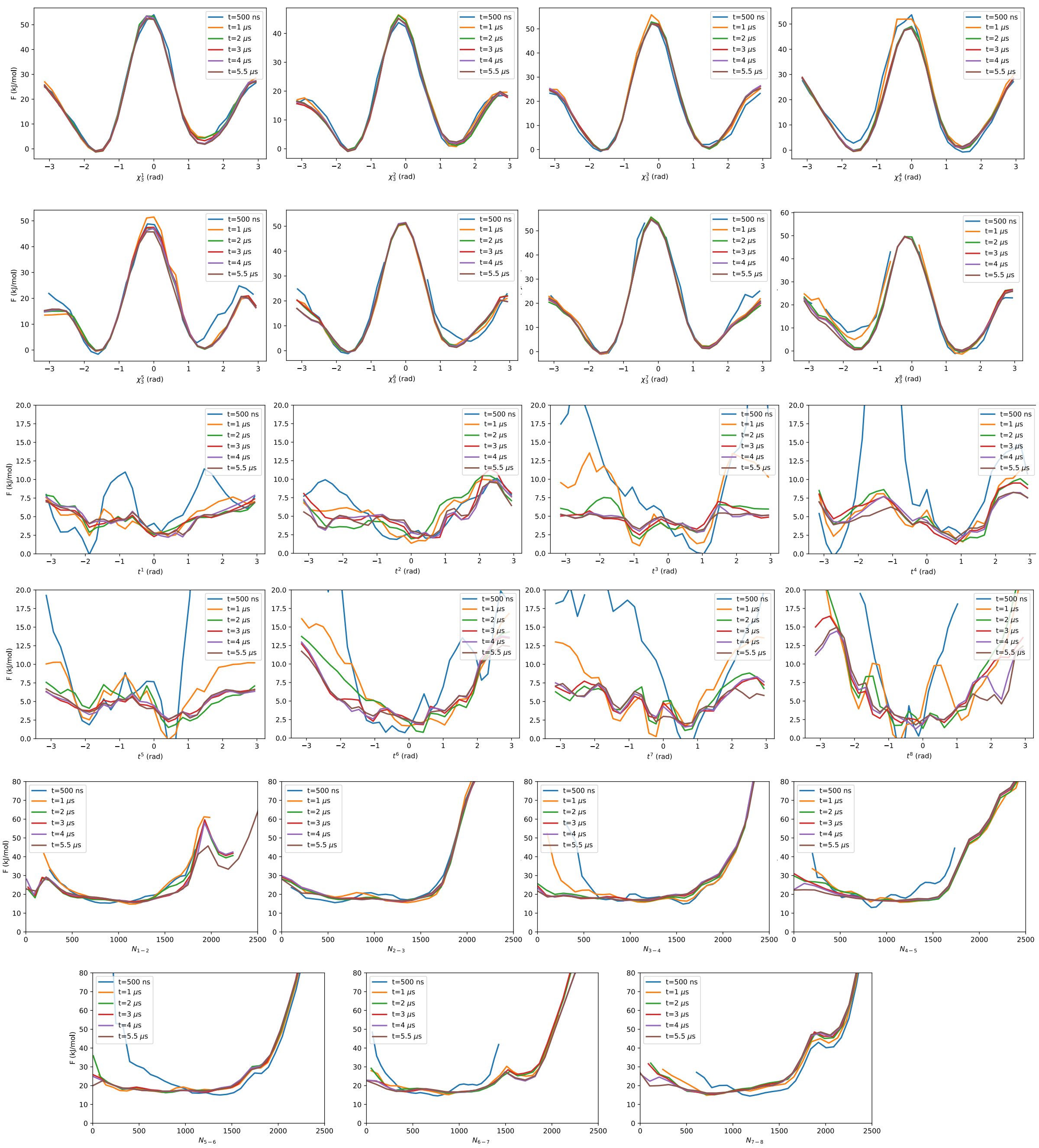

Figure S3. Assessment of the convergence of the MEMMI simulation. Free energy profiles for all biased collective variables for subsequent 1 μs increments of simulated time.

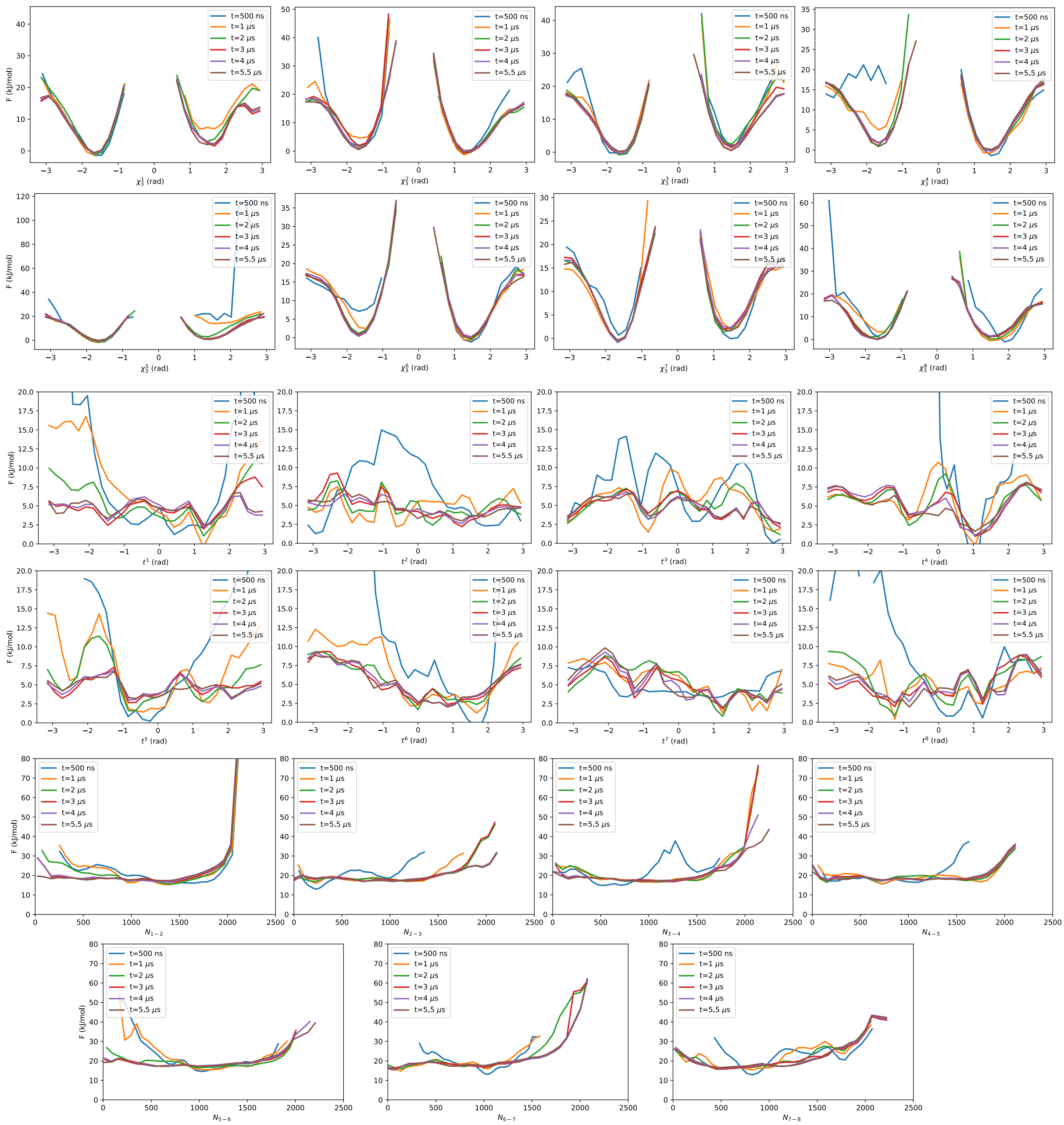

Figure S4. Assessment of the convergence of the EMMI simulation. Free energy profiles for all unbiased collective variables for subsequent 1 μs increments of simulated time.

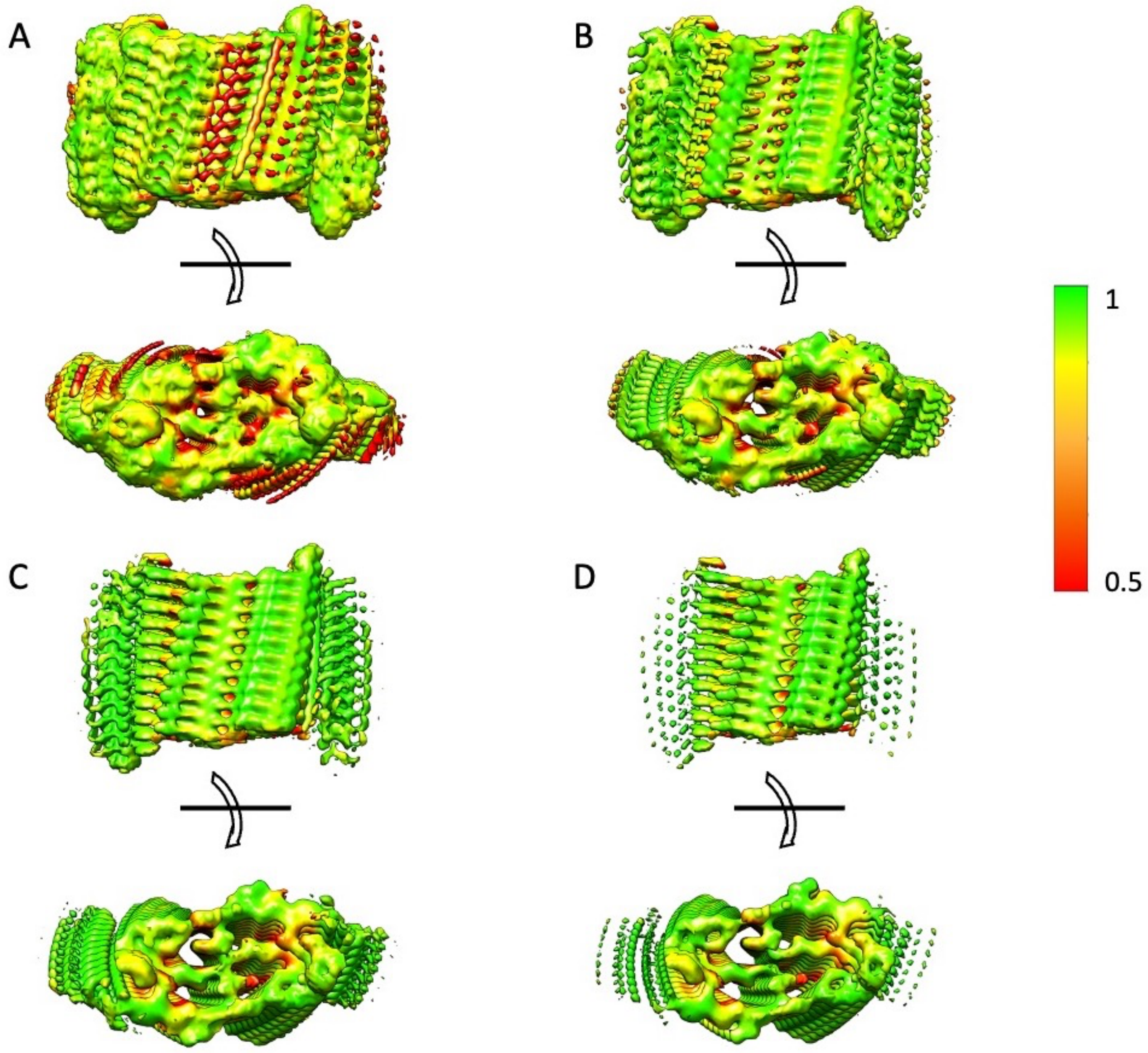

Figure S5. Assessment of the local correlation between experimental and calculated cryo-EM map. Local correlation of the cryo-EM map (EMD-EM-10669) with a map generated from the MEMMI ensemble of an IAPP amyloid fibril as a function of increasing strength of density (decreasing electron density thresholds): 1σ (A), 2σ (B), 3σ (C), and 4σ (D).

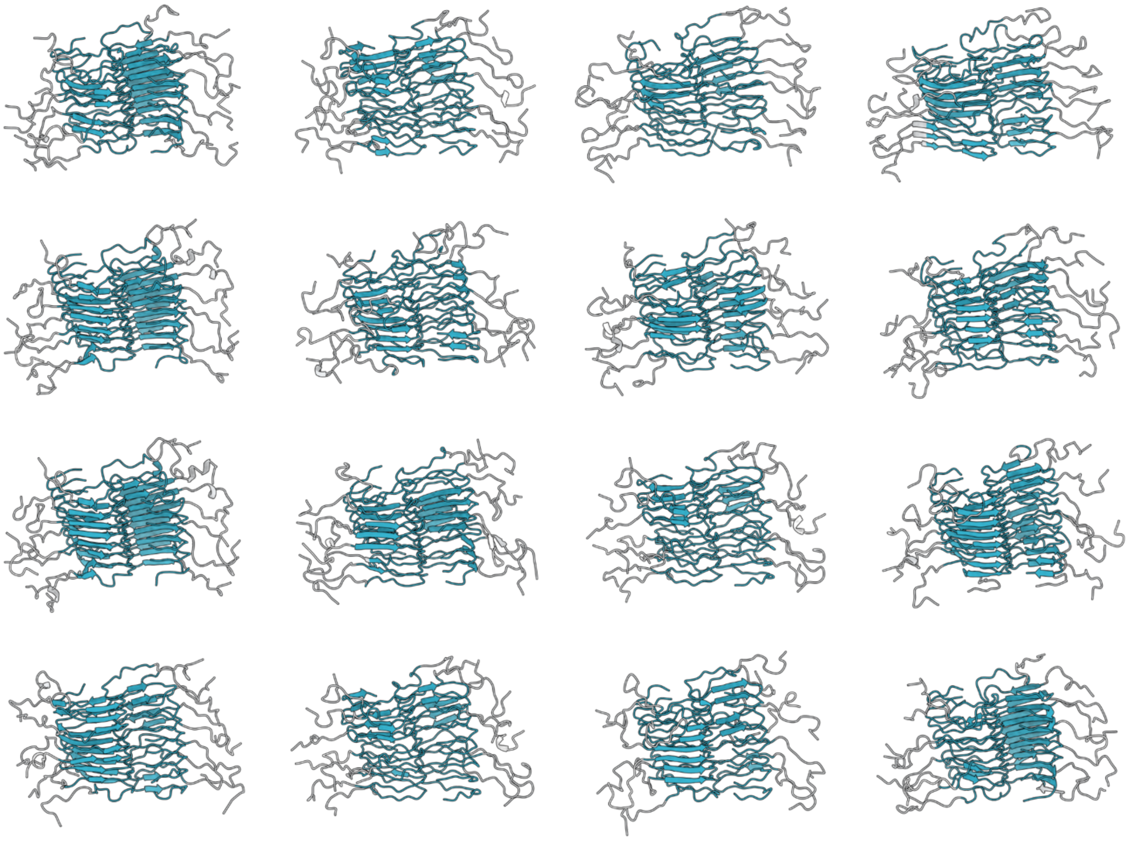

Figure S6. Individual conformations of the final structural ensemble. Sample of 16 structures from the ensemble shown in **Figure 2**.

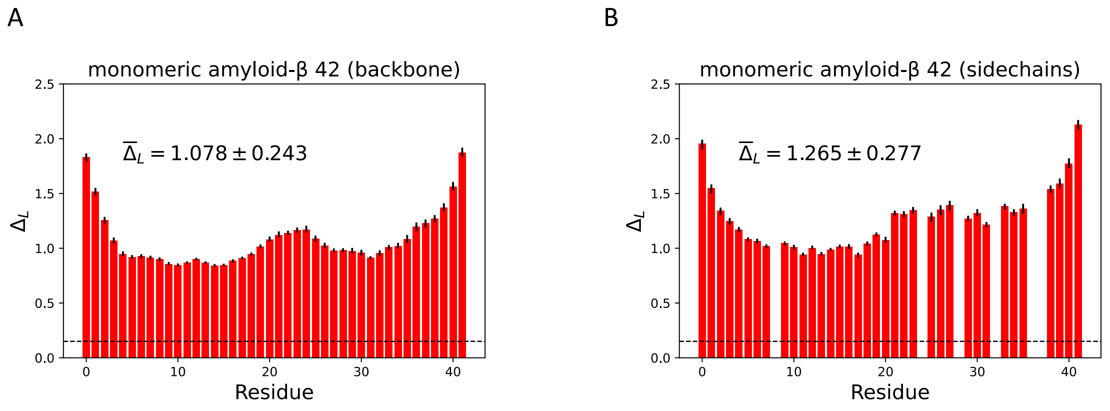

Figure S7. Lindemann parameters for the disordered monomeric amyloid-β 42 peptide computed from a previously published ensemble[27]. Lindemann parameters calculated for the backbone **(A)** and side chains **(B)** with the liquid-solid transition and residue-mean indicated. Error bars indicate the 95th percentile of the mean of a bootstrap sample over all 5119 trajectories in the ensemble.
